# Role of Electrically Evoked Muscle Hypertrophy on Spasticity in Persons with Spinal Cord Injury

**DOI:** 10.3390/jcm14113972

**Published:** 2025-06-04

**Authors:** Momal A. Wasim, Ahmad M. Alazzam, Ashraf S. Gorgey

**Affiliations:** 1 Spinal Cord Injury and Disorders Center, Richmond VA Medical Center, Richmond, VA 23249, USA; momal.hashmi@va.gov (M.A.W.); ahmadalazzam6@gmail.com (A.M.A.); 2 Department of Physical Medicine and Rehabilitation, Virginia Commonwealth University, Richmond, VA 23298, USA

**Keywords:** spasticity, neuromuscular electrical stimulation, resistance training, testosterone, spinal cord injury

## Abstract

**Study Design:** Pilot randomized clinical trial. **Objective**: To examine the effect of electrically evoked muscle hypertrophy on indices of spasticity, as measured by Biodex after spinal cord injury (SCI). **Setting**: Medical research center. **Methods**: Thirteen males with chronic SCI were randomized into sixteen weeks of either surface neuromuscular resistance training (NMES-RT) + testosterone treatment (TT) (n = 7) or a TT-only group (n = 6). A Biodex isokinetic dynamometer was used to measure knee extensor and flexor muscle spasticity at the beginning (baseline; BL) and at the end (post-intervention; PI) of 16 weeks. The passive tension of the right knee extensor and flexor muscle groups were evaluated at angles of 5°, 30°, 60°, 90°, 180°, and 270° per second (sec). Dual energy X-ray absorptiometry and magnetic resonance imaging were used to measure leg lean mass and thigh muscle cross-sectional areas (CSAs). **Results**: Robust muscle hypertrophy was noted in leg lean mass [11%, *p* = 0.023] as well as whole thigh [17%, *p* = 0.001] and knee extensor muscle [28%, *p* = 0.001] CSAs in the NMES-RT+TT compared to the TT-only group. There was no difference in extensor or flexor spasticity between the NMES-RT+TT or TT-only groups at different angular velocities following 16 weeks of intervention. Collapsing the extensor passive torques indicated an (24–28%) increase (*p* < 0.004) in response to angular velocities at BL and following PI measurements [180 deg/sec (23%; *p* = 0.03) and 270 deg/sec (32%; *p* = 0.009)] compared to 5 deg/sec. The extensor slope showed a non-significant (*p* > 0.05) decrease of 15–28% across all angular velocities. The catch-AB slopes were non-significantly lower in the TT-only group compared to the NMES-RT+TT at higher speeds [90 deg/sec and 270 deg/sec] and attained a trend towards lower passive torque at 180 deg/sec [180 deg/sec: 15.5%, *p* = 0.05]. **Conclusions**: Evoking skeletal muscle hypertrophy did not increase spasticity indices at different angular velocities following sixteen weeks of NMES-RT+TT or TT in persons with chronic SCI. Augmenting muscle hypertrophy is likely to attenuate the hyper reflexive slope of the extensor spasticity. The findings may suggest that evoking muscle hypertrophy following NMES-RT does not increase indices of spasticity after SCI. The clinical implications are highly important in managing spasticity after SCI.

## 1. Introduction

Spinal Cord Injury (SCI) is considered a life-altering injury with an annual incidence of approximately 54 cases per one million people in the United States, or about 18,000 new SCI cases every year [1,2,3]. Persons with SCI suffer from sequential and detrimental neuromuscular and cardio-metabolic changes characterized by muscle atrophy within weeks to months post-injury, an altered body composition and increased ectopic adiposity [4,5,6,7,8]. These detrimental changes primarily originate from insult to the spinal cord that results in extreme disuse [7,8], unloading and a reduction in the level of physical activity [9]. Individuals with SCI may subsequently develop a myriad of cardiometabolic comorbidities including premature aging, mitochondrial dysfunction, impaired glucose tolerance, insulin resistance and dyslipidemia [10,11].

Over the last two decades, there has been a growing interest in reversing the aforementioned profile, restoring lean mass and evoking muscle hypertrophy [12,13,14]. A key hypothesis is that restoring muscle size could enhance the paracrine capability of the muscle to mitigate several cardiometabolic comorbidities after SCI [15,16]. Additionally, restoring muscle size is likely to decrease the infiltrated ectopic intramuscular fat and visceral adiposity, increase the disposal of circulating plasma glucose, and enhance bone health after SCI [17]. Resistance training (RT) has been shown to boost patients’ anabolic profile to restore lean mass [18,19,20], increase the muscle cross-sectional area (CSA) [21,22] and reduce aging related cardio-metabolic disorders in several other clinical populations [18,19,20]. In persons with SCI, electrically evoked RT via the application of surface neuromuscular electrical stimulation (NMES) has been characterized as the most successful rehabilitation intervention in the restoration of muscle size [12,21,22]. Several studies have demonstrated that NMES-RT successfully evokes muscle hypertrophy by up to 35–40% following 12–16 weeks of training in persons with SCI [12,21]. Additionally, studies have shown that NMES-RT in combination with testosterone treatment (TT) is likely to enhance muscle quality; this is characterized by increasing the CSA of the trained and untrained muscle groups and increasing the peak isometric and isokinetic torques, as well as the specific tension [6,22].

A controversial unanswered question is whether electrically evoked muscle hypertrophy may alter the level of spasticity in persons with SCI. For example, 65% to 78% of individuals with SCI commonly experience symptoms of spasticity within the first year post-injury [23,24]. Spasticity is a symptom resulting from upper motor neuron injury and is a motor disorder characterized primarily by a velocity-dependent increase in tonic stretch reflexes (muscle tone), with hyperreflexia and exaggerated tendon jerks [25]. In addition, spasticity involves more than velocity-dependent resistance, encompassing the development of muscle spasms, hyperreflexia and sustained contractions [26]. A previous clinical randomized trial indicated that 12 weeks of RT increased the strength of partially paralyzed muscles without demonstrating deleterious effects on spasticity during acute inpatient rehabilitation in persons with SCI [27]. These are highly important findings considering the recognized negative consequences of spasticity within the clinical and research communities [19,20,23,24]. Spasticity is likely to impact patients’ quality of life, decrease their range of motion and contribute to pressure injuries and the development of urinary tract infection after SCI. If left untreated, spasticity may lead to the development of musculoskeletal contractures and reduce independency after SCI [14,15,28,29]. Therefore, the purpose of the current work was to examine whether electrically evoked muscle hypertrophy via the application of NMES-RT would increase knee extensor or flexor spasticity in individuals with chronic SCI.

## 2. Methods

Thirteen male participants were previously enrolled in a sixteen-week randomized clinical trial to examine the effects of NMES-RT+TT compared to TT only on cardiometabolic risk factors after SCI [12]. After four weeks of delayed entry, participants were randomly assigned into either the NMES-RT+TT group (n = 7) or TT group (n = 6). The design and the primary outcome variables of the trial were previously published [12]. The study received approval from a local ethical committee and was registered on clinicaltrials.gov (NCT01652040). A research team member explained the study and obtained verbal and written informed consent from all participants. All aspects of the study procedures were conducted according to the Declaration of Helsinki. This is followed by a comprehensive physical examination by a board-certified physiatrist in SCI medicine. The examination included a neurological assessment based on the International Standards for Neurological Classification of SCI (ISNCSCI) and the American Spinal Injury Association (ASIA) Impairment Scale (AIS) [1].

## 3. Study Design

Briefly, transdermal TT patches (2–6 mg/day) were administered daily for 16 weeks in both groups. The NMES-RT+TT group underwent 16 weeks of supervised progressive RT via the application of surface NMES and ankle weights. Spasticity measurements were conducted at the beginning (baseline; BL) and at the end of the 16 weeks (post-intervention; PI). Participants were men between 18 and 50 years of age (Table 1).

The upper age limit was selected to minimize the risk of unanticipated side effects associated with TT. All participants had been diagnosed with complete motor SCI between levels C5 and L2, classified as ISNCSCI Impairment Scale A or B. Individuals with pre-existing medical conditions that could interfere with the intervention were excluded. These included cardiovascular disease, uncontrolled type II diabetes or current insulin use, stage 2 or greater pressure injuries, supra-physiological testosterone levels, hematocrit above 50%, and active urinary tract infections or associated symptoms. The inclusion and exclusion criteria of the trial were previously listed in detail [12,22].

### 3.1. Intervention

#### 3.1.1. NMES- Resistance Training (RT)

NMES-RT was conducted twice weekly for 16 weeks [6,12]. During the first week of training, participants were instructed not to lift ankle weights to establish the full range of motion in their knee against gravity. This ensured that the knee extensor muscle could move the weight of the lower limb against gravity. The training program consisted of 4 sets of 10 repetitions (4 × 10 reps) that started with the right leg (1 set × 10 reps) and alternated with the left leg (1 set × 10 reps) until completion of the 4 sets per leg (total 40 reps per leg). A period of 2–3 min of rest was allowed between sets in the right and left legs.

Surface NMES was applied to the knee extensor muscles using adhesive gel electrodes. One electrode was placed 2–3 cm above the superior aspect of the patella over the vastus medialis muscle, and the other electrode was positioned lateral to and 30 cm above the patella over the vastus lateralis muscle. The current from the stimulator was manually ramped up in 5 s intervals to achieve full knee extension. The stimulation parameters were adjusted at 30 Hz with a rectangular biphasic pulse duration of 450 μs and a current amplitude sufficient to evoke full knee extension [12,22].

Once full knee extension was achieved against gravity, commercially available ankle weights were progressed in increments of 2 lbs. on a weekly basis. The progression of ankle weights was determined based on the ability of the knee extensor muscle group to perform 4 sets × 10 reps. If the knee extensor muscles failed to perform 4 sets × 10 reps, the same weight was maintained for the following week until the desired goal was achieved. All training procedures were supervised and completed by a trained research staff while all participants were seated in wheelchairs [12,21,22].

#### 3.1.2. Testosterone Treatment (TT)

Following BL measurements, transdermal testosterone was administered using Androderm patches (Watson Pharma, Parsippany, NJ, USA), delivering between 2 and 6 mg/day in both groups [12,22]. Participants were instructed to apply the patches at bedtime and only remove them for showering. The serum testosterone (T) concentration was measured and reviewed weekly in a blinded fashion for the first month and then every four weeks by an endocrinologist. The initial T dose was based on the baseline T level: 6 mg/day if <300 ng/dL, 4 mg/day if 300–600 ng/dL, and 2 mg/day if >600 ng/dL. If the serum T concentration exceeded 1000 ng/dL (34.7 nmol/L) during the study, the dose was reduced to 2 mg/day. If the concentration was <250 ng/dL (8.7 nmol/L) above the pre-treatment level, the participants were re-educated on patch usage. Used patches were returned and counted to ensure adherence to the protocol.

### 3.2. Measurements

#### 3.2.1. Body Weight and Height

A wheelchair scale (Tanita, Arlington Heights, IL, USA) was used to measure the weight of participants. Participants were placed in a supine lying position when measuring height (m). The soles of the feet were placed against a board to ensure the neutral positioning of both feet. Height was measured using a Harpenden Stadiometer to the nearest 0.1 cm. Body mass index (kg/m^2^) was then calculated as weight (kg) divided by height (m^2^) [30].

#### 3.2.2. Lean Mass Measured by DXA

Leg lean mass was measured by the General Electric Lunar DXA scanner (GE Lunar Inc, Madison, WI, USA). The same technician obtained and analyzed DXA scans at BL and PI to primarily determine the leg lean mass for the purpose of the current study. After calibration, all participants were placed in a supine lying position on the scanning table with arms internally rotated and palms medially facing. A total body scan was conducted and only leg lean mass was used for the current trial. In persons with SCI, the coefficient of variabilities for repeated scans was determined as previously established [30].

#### 3.2.3. Whole Thigh and Knee Extensor Cross-Sectional Areas (KE-CSA)

Magnetic resonance imaging (MRI) of the right whole thigh was performed and the KE muscle CSAs were measured before (BL) and after the 16-week interventions (PI) using a 1.5 Tesla GE magnet [fast spin echo; repetition time, 850–1000 ms; echo time, 6.7 ms; imaging frequency, 63.8 MHz; echo number, 1; echo train length, 3; flip angle, 90°; field of view, 20 cm; matrix size, 256 × 256]. Approximately 12–15 transaxial images, 0.8 cm thick and 1.6 cm apart, were captured in a supine lying position from the hip to the knee joint using a General Electric body array flex coil to measure thigh CSA. MRI analysis was performed using WinVessel 2.011-vessel (East Lansing, MI, USA). The software was then used to calculate the whole thigh and knee extensor CSAs (KE CSA) for each slice. The average of 12–15 slices was considered as a measure of the whole thigh and KE CSA [12,21].

#### 3.2.4. Spasticity Measurements

The passive torques of the right knee extensor and flexor muscle groups were evaluated using a Biodex isokinetic dynamometer (Shirley, New York, NY, USA) and the data were collected on the Lab Chart 8 application [21,22]. Measurements were collected at BL prior to the 16-week interventions and at 4–5 days at the end of both interventions (PI). Participants were seated with their trunk–thigh angle and knee–thigh angle set at 90°. They were securely strapped to the test chair using crossover shoulder harnesses and a hip joint belt. The dynamometer axis was aligned with the anatomical knee axis, and the lever arm was attached 2–3 cm above the lateral malleolus. The passive tension in the right knee extensor and flexor muscle group was assessed at angles of 5°, 30°, 60°, 90°, 180°, and 270° per second to measure spasticity levels [31,32].

Movement of the leg into extension reflected the passive tension of the flexor muscle group (i.e., hamstring muscles); meanwhile, movement of the leg into flexion reflected the passive tension of the knee extensor muscle group. The range of motion of the dynamometer was adjusted to allow knee extension arc from 90 deg flexion to 5 deg extension (0 deg reflects 180 deg knee extension) and knee flexion arc from 175 deg to approximately 70 deg. All passive movements were introduced twice in a seated position at different angular velocities [31,32].

**Figure 1:**
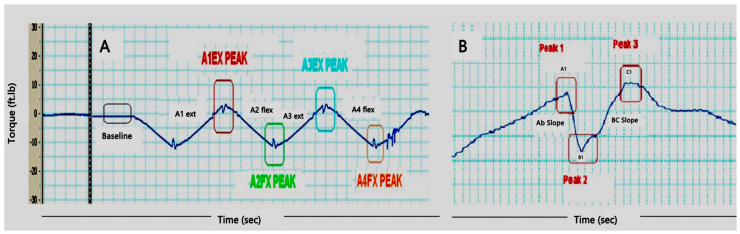
Representative diagram demonstrating the step-by-step procedure of analyzing the extensor and flexor spasticity in persons with SCI. (**A**) A1 ext represents the movement of the Biodex arm into flexion direction to measure the passive extension torque (A1 ext torque) and the slope (A1 ext slope) and ends A1Ex peak; A2 flex represents the movement of the biodex arm into the extension direction to measure the flexion torque (A2 flex torque) and flexion slope (A2 flex slope) and ends at A2 flex peak. The pattern was then repeated for A3 ext and A4 flex, respectively. The A1 and A3 data were then averaged to provide the extensor torques and extensor slopes at different angular velocities. A2 and A4 were also averaged to provide the flexor torques and flexor slopes at different angular velocities. The A1EX and AIEX3 peaks represent the catch and release at the end of the extensor pattern. (**B**) Represents the zoomed-in pattern of the catch (AB-slope) and release (BC-slope) analysis. After magnifying the catch and release, three major peaks were identified as A1, B1 and C1 and the slope between A1 and B1 (catch), followed by the slope between B1 and C1 (release), was measured.

Passive torque data were collected during the BL and PI measurements for participants in the NMES-RT+TT and TT-only groups. To evaluate spasticity, passive torque was assessed for the extensor and flexor muscle groups separately. The data were captured and analyzed using Lab Chart 8. The mean torque (Nm) and slope (Nm/s) of the knee extensors and flexors were calculated as highlighted in Figure 1. The mean torque represents the magnitude of the spasticity, and the slope represents the level of hyperexcitability of the spasticity. The mean torque was calculated after subtracting the baseline value, as indicated in Figure 1.

Each participant performed two repetitions of extensor activity, A1-extension and A3-extension, and two repetitions of flexor activity, A2-flexion and A4-flexion for every rotational speed (Figure 1). A1 and A3 were averaged to reflect extensor spasticity, and the data for A2 and A4 were also averaged to measure knee flexor spasticity.

**Figure 2:**
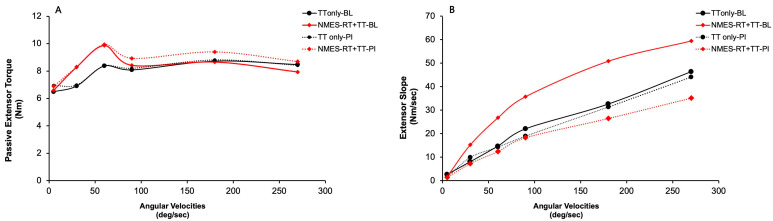
Passive extensor torque (**A**) and slopes (**B**) at baseline (BL; solid red and black lines) and post-intervention (PI; red and black dashed lines). TT only-BL (solid black line), TT only-P1 (dashed black line), NMES-RT+TT-BL (solid red line), and NMES-RT+TT-P1 (dashed red line) are indicated for each group. Standard deviations were omitted for clarity.

The slopes of the catch (AB-slopes) and release (BC-slopes) of the extensor knee muscle groups were also evaluated at the end of different knee angular velocities. Catch and release, according to the Ashworth scale, refers to the minimal resistance encountered towards the end of the range of motion [33]. The catch and release were identified as the change in the direction of the reported torque before returning to the original direction, as denoted in Figure 2.

Each slope direction was measured twice and then the average was obtained. Finally, muscle spasticity was also evaluated using the Modified Ashworth Scale. Anti-spasmodic medications were also reported for all study participants in both groups (Table 2).

#### 3.2.5. Statistical Analyses

All data were primarily checked for normality using Shapiro–Wilk tests. Q-Q plots were used to visually detect outliers and outliers were omitted to assume normality. Univariate analysis of covariance (ANCOVA) was used to determine the difference between the NMES-RT+TT and TT-only groups at PI after controlling for BL measurements. This was followed by a mixed-model analysis of variance (ANOVA) to determine the within-group effect (i.e., time effect) or interaction between groups. Data were presented as mean ± SD and statistical significance was set at a level of less than 0.05. Analysis was completed using IBM-SPSS (version 29.0, SPSS, Chicago, IL, USA).

## 4. Results

The physical characteristics of participants with SCI are presented in Table 1. Independent *t*-tests indicated that were no difference in the physical or SCI characteristics between both groups. The Modified Ashworth scores with antispastic medications are listed in Table 2. Participants did not report changes in the dose of antispastic medications throughout the trial.

### 4.1. Muscle Hypertrophy

In summary, DXA-leg lean mass increased by 11% in the NMES-RT+TT [BL: 14.7 ± 4.1 and P1: 16.4 ± 3.5 kg; *p* = 0.023] without any changes in the TT-only group [BL: 13.0 ± 1.4 and PI: 12.7 ± 1.2 kg; *p* = 0.75]. Mean differences indicated grater changes in leg lean mass in the NMES-RT+TT compared to TT-only groups [1.7 ± 1.3 vs. −0.26 ± 0.76 kg; *p* = 0.013].

Whole the thigh muscle CSA [BL: 107 ± 30.5 and PI: 125.2 ± 31.5 cm^2^; *p* = 0.001] and knee extensor [BL: 47 ± 13 and PI: 60.0 ± 12.6 cm^2^; *p* = 0.001] CSAs showed 17% and 28% increases, respectively, in the NMES-RT+TT without changes in the TT-only group, the mean differences indicated a greater whole thigh muscle CSA in the NMES-RT+TT group compared to the TT-only group [18 ± 8.2 vs. 2.5 ± 7.6 cm^2^; *p* = 0.0005]. PI measurements indicated that the whole KE muscle CSA was 39% greater in the NMES-RT+TT group compared to the TT-only group [60.0 ± 12.6 vs. 43 ± 15.0 cm^2^; *p* = 0.048].

### 4.2. Extensor Spasticity

Figure 2 presents the mean extensor spasticity torque at different angular velocities following either NMES-RT+TT or TT only at BL and PI. The results indicated that there were no differences between both groups at different angular velocities [5 deg/sec (*p* = 0.25; *ηp^2^* = 0.14); 30 deg/sec (*p* = 0.6; *ηp^2^* = 0.027); 60 deg/sec (*p* = 0.8; *ηp^2^* = 0.005); 90 deg/sec (*p* = 0.62; *ηp^2^* = 0.029); 180 deg/sec (*p* = 0.8; *ηp^2^* = 0.008) and 270 deg/sec (*p* = 0.22; *ηp^2^* = 0.18)]. To examine the time effect, repeated measures ANOVA indicated that there was a non-significant decrease in passive torques (*p* = 0.2–0.8) in the NMES-RT+TT group from BL to PI [−4% at 60 deg/sec −16% at 270 deg/sec], with insignificant changes in the TT-only group (see Supplementary Table S1).

BL measurements indicated increases in passive torques (*p* < 0.004) at different angular velocities [60 deg/sec (37%; *p* = 0.014); 90 deg/sec (28%; *p* = 0.009); 180 deg/sec (38%; *p* = 0.007) and 270 deg/sec (24%; *p* = 0.03)] and at PI measurements [180 deg/sec (23%; *p* = 0.03) and 270 deg/sec (32%; *p* = 0.009)] compared to 5 deg/sec after collapsing the extensor spasticity data from both groups.

Figure 2 presents the slope of the extensor spasticity; the results indicated that were no differences between both groups at different angular velocities *(p* = 0.4–0.7). Furthermore, the rate of rise in the slope of extensor spasticity did not change over time in both groups *(p* = 0.4–0.7). The rate of rise in extensor spasticity significantly increased at different angular velocities after collapsing the data between both groups at BL (*p* = 0.001) and PI (*p* = 0.001). At BL, pairwise comparisons indicated that the extensor spasticity slope increased at 90 deg/sec (*p* = 0.06), 180 deg/sec (*p* = 0.02) and 270 deg/sec (*p* = 0.04) compared to 5 deg/sec. At P1, pairwise comparisons indicated that the extensor spasticity slope increased at all angular velocities (30 deg/sec −270 deg/sec; *p* = 0.0001) compared to 5 deg/sec.

After collapsing the data, the extensor slope showed a non-significant (*p* > 0.05) decrease from 15–28% across all angular velocities in PI compared to BL.

### 4.3. Flexor Spasticity

There were no differences in the flexor spasticity torques (*p* = 0.4–0.8; *ηp^2^* = 0.006–0.025) or slopes (*p* = 0.4–0.9) between groups (NMES-RT+TT and TT only) at different angular velocities in PI. Furthermore, there were no time effects in flexor torques (*p* = 0.1–0.8) or slopes (*p* =0.1–0.8) following either intervention (see Supplementary Table S1).

After collapsing the data from both groups, the flexor spasticity torques decreased (*p* = 0.0001) at BL [60 deg/sec (30%; *p* = 0.04); 90 deg/sec (33%; *p* = 0.047); 180 deg/sec (37%; *p* = 0.005) and 270 deg/sec (39%; *p* = 0.002)] and PI [180 deg/sec (30%; *p* = 0.003) and 270 deg/sec (37%; *p* = 0.001)] relative to 5 deg/sec. Finally, the flexor slopes increased at different angular velocities at BL (*p* < 0.0001) and PI (*p* < 0.0001) relative to 5 deg/sec.

### 4.4. Catch and Release Slopes

There were no differences in the extensor release slopes between both groups. Univariate ANCOVA indicated that the catch-AB slopes were non-significantly lower in the TT-only group compared to the NMES-RT+TT at higher speeds [90 deg/sec: 5.5%, *p* = 0.46; 270 deg/sec: 12.5%, *p* = 0.62] but only attained a trend towards statistical significance at 180 deg/sec [180 deg/sec: 15.5%, *p* = 0.05].

## 5. Discussion

The major goal of the current study was to examine the effects of electrically evoked muscle hypertrophy induced by NMES-RT+TT on velocity-dependent knee extensor and flexor spasticity in persons with chronic SCI. Previous findings demonstrated significant muscle hypertrophy, specifically showing an increase in the whole thigh muscle CSA by 28–34% and knee extensor CSA by 31–34% following NMES-RT +TT [12]. A follow-up study indicated that muscle hypertrophy is extended to include the total glutei (8–11%), gluteus maximus (14–16%) and gluteus medius muscles (10–14%) [22]. Given these findings, an initial hypothesis proposed that muscle hypertrophy induced by NMES-RT +TT may concurrently result in increased extensor–flexor muscle spasticity. In contrast, our results demonstrated that knee extensor and flexor spasticity remained statistically insignificant between the NMES-RT+TT and TT-only groups. Furthermore, we observed a non-significant decline in the slope of the extensor torque–velocity curve following NMES-RT+TT [Figure 2B], suggesting possible beneficial effects rather than an adverse increase in muscle spasticity.

### 5.1. Rationale of the Study

Previous reports have shown that RT has no deleterious effects on spasticity in neurologic populations [27]. However, evoking muscle hypertrophy is accompanied by an increase in the CSA of both extrafusal and intrafusal muscle fibers [34,35]. The increase in the size of the intrafusal muscle fibers is likely to be accompanied by an increase in the afferent firings of Ia and II fibers; this may subsequently lead to increases in the mono-synaptic stretch reflex and muscle spasticity [36]. Furthermore, heightened reflex activity from larger intrafusal fibers may necessitate higher doses of anti-spastic medications such as oral baclofen [37]. This hypothesis may be in line with the unsupported clinical claim that strength training may negatively provoke spasticity in persons with SCI.

Considering mobility impairment and patient-reported spasticity, our findings are clinically significant to the SCI community. None of our participants were required to increase the dose of the anti-spastic medications administered during the 16-week intervention. This may highlight that the proposed interventions, either NMES-RT+TT or TT only, did not clinically elicit changes in indices of spasticity after SCI. This is of clinical importance considering the well-established side effects of anti-spastic medications [38,39]. Additionally, dosages of oral baclofen or anti-spastic medications could be clinically used to gauge the effect of any rehabilitation intervention on spasticity. Furthermore, muscle hypertrophy in the current trial was accompanied by a significant increase in body mass index (BMI) from 25.0 to 26.6 kg/m^2^ following NMES-RT+TT [12,22]. It was previously established that increases in BMI may necessitate an increase in the dosage of oral baclofen [37]. This study pinned the possibility that oral baclofen may diminish the protective effects of spasticity on patients’ muscle size and metabolic profile after SCI [37]. However, the findings did not support the hypothesis that oral baclofen dosages may negatively influence metabolic or body composition parameters in persons with SCI [37]. On the contrary, a previous case report indicated that 20 years of intrathecal baclofen may adversely affect musculoskeletal health and accelerate bone resorption at the distal femur and proximal tibia compared to age-matched individuals [39]. Importantly, these findings emphasize the importance of investigating alternative treatments for spasticity management that minimize long-term reliance on pharmacological interventions. Finally, it further highlights the significance of adding electrically evoked RT in conjunction with anti-spastic medications to attenuate any negative effects on musculoskeletal health after SCI. Therefore, rehabilitation strategies that promote muscle hypertrophy or enhance anabolic profile are considered sufficiently clinically acceptable to be integrated into the management of persons with SCI.

### 5.2. Use of Isokinetic Dynamometer to Objectively Measure Spasticity

Previous trials have used the Modified Ashworth scale (MAS) to subjectively evaluate spasticity [40,41]. However, the current trial used an isokinetic dynamometer to objectively measure spasticity via the implementation of passive mode at different velocities. Objective measurements are critical in the research environment because clinical spasticity scales may be insensitive to subtle changes and be influenced by the examiner’s technique. For example, a previous systematic review highlighted the temporary effects of electrical stimulation exercise on spasticity after SCI [42]. A lack of objective measurements may explain the controversial speculations about failing to recognize the long-term or carryover effects of training on spasticity [42].

We previously evaluated extensor–flexor spasticity relative to locomotor training in persons with SCI [31]. Others have successfully developed a portable system to measure knee extensor spasticity by relying on the pendulum test and inertial sensors that were placed on the iliotibial band and the mid-part of the lower leg [43]. The findings indicated excellent agreement with the optical tracking system in persons with SCI [43]. Moreover, a velocity-dependent increase in passive torques was observed with increasing angular velocities in our baseline assessment [25]. Recent research has recognized spasticity as a potential biomarker of motor recovery [44]. Sangri et al. evaluated sixty-six participants with sub-acute SCI; pendulum tests and modified Ashworth scales were administered for both motor complete and incomplete SCI [44]. Motor-evoked potentials were also measured via transcranial magnetic stimulation to activate the rectus femoris muscle. The findings showed that 36% of the examined sample with spasticity demonstrated improvements in motor and sensory scores at the time of discharge from inpatient rehabilitation [44]. The authors suggested that spasticity may serve as a promising neurophysiological biomarker to indicate recovery in persons with subacute SCI [44]. The findings align with prior evidence suggesting that spasticity may play a neuroprotective role in mitigating muscle atrophy and bone loss after SCI [40,45]. Moreover, spastic muscle activity may confer metabolic or musculoskeletal benefits following SCI. Previous works have indicated that individuals with more severe spasticity are likely to maintain fat-free mass, which is important for the maintenance of the basal metabolic rate [40]. A diminished basal metabolic rate and increased caloric consumption have been primarily considered as the root cause of the high prevalence of obesity after SCI [46]. Severe spasticity is associated with decreased adiposity and fasting plasma glucose [47]. The neuroprotective effect of spasticity on muscle mass is mediated via circulating insulin growth factors (IGFs) [48].

The captured variables represent important neurophysiological components of the spasticity paradigm after SCI. The slope of the curve reflects the hyperexcitability of the stretch reflex, more specifically the rate of excitability of spinal and inter-spinal neurons. The rate of rise in a slope is indicative of faster stretch reflex activity via the movement of the limb in the opposite direction (i.e., flexion of the knee joint to elicit extensor spasticity) at different angular velocities relative to 5 degrees per second. The extensor slope demonstrated a non-significant decrease (~15–28%) after pooling both groups, which may suggest the decreasing hyperexcitability of the extensor stretch reflex. Additionally, the calculated standard deviations suggest a decrease in the variability of the extensor stretch reflex component and the conversion of the heterogenous nature of the extensor spasticity before interventions into a more homogenous muscle tone following 16 weeks.

### 5.3. Potential Mechanism of NMES-RT or TT on Spasticity

Both groups received anabolic hormone therapy, which allowed us to examine the specific contributions of TT with regard to neuromuscular plasticity and spasticity independent of exercise. At higher stretch velocities, the TT-only group demonstrated a reduction in the catch angle slope, which raises the possibility that testosterone may exert an anti-spastic or neuroprotective effect. The influence of muscle hypertrophy following NMES-RT+TT or TT on spasticity is still controversial. A potential mechanism is mediated via the impact on the plateau potentials of spinal motor neurons. Plateau potentials are considered persistent voltage-gated sustained periods of depolarization that increase hypertonicity without continuous input, which are exaggerated following SCI and contribute to long reflex contractions and muscle spasms [49]. A reduction in plateau potentials could dampen hypertonicity and spastic responses [50].

Additionally, previous animal studies have shown that testosterone has a neuroprotective effect on spinal neurons [51,52]. For instance, Byers et al. showed that in a T9 contusion rat model, four weeks of TT preserved the dendritic length of injured quadriceps motoneurons, in addition to attenuating the atrophy of muscle fibers innervated by these neurons [52]. TT attenuated motor neuron loss, which is typically associated with muscle atrophy and spasticity as a result of disuse [51]. Approximately 35–43% of individuals with SCI suffered from decreased circulating testosterone levels and the development of hypogonadism [53,54]. Therefore, administering TT with or without NMES-RT may increase the neuroprotective effects on the spinal motor neuron pools and decrease the slope of extensor spasticity. Although the exact mechanism warrants further research, our findings may suggest an independent effect of TT on the hyperactive stretch reflex. Importantly, anabolic hormone therapy may decrease spasticity while also improving muscle quality and bone health after SCI.

### 5.4. The Catch and Release

The catch and release slopes of the extensor muscles in response to fast stretch were measured in both groups. This analytical approach was previously adopted in children with cerebral palsy [33]. In clinical settings, the catch is commonly described as a sudden increase in the resistance, which interferes with the range of motion in response to fast passive stretch. The catch is commonly associated with a sudden increase in muscle activity, as determined by EMG and the deceleration of the joint angle [33]. In addition, the catch slope represents the steepness of the torque rise at the stretch onset. The release slope indicates the decline of torque as the muscle lengthens. The use of TT only decreased the magnitude of the catch slopes at higher knee angular velocities. The decrease in the catch slopes occurred despite the limited muscle hypertrophy in the TT-only group compared to the NMES-RT+TT group. The exact mechanism behind this observation remains unclear and may suggest the possible independent effects of TT on the hyperactive stretch reflex in persons with SCI.

The current findings ensured that we could objectively measure the components of the catch and release in response to different angular velocities. Importantly, our findings suggest that different components of spasticity (phasic vs. tonic, catch vs. continuous resistance) may respond differently to various interventions. Such components are integral parts of the definition of the spasticity complex and should be considered in the underlying pathology and management of spasticity after SCI. Our methodology provides a framework to assess spasticity with greater precision than conventional subjective rating scales. This may facilitate the development of more targeted interventions that address distinct components of spasticity after SCI.

### 5.5. Limitations

The current study was conducted primarily to examine the effects of combing NMES-RT and TT on cardiometabolic risk factors after SCI. Spasticity was measured as a secondary outcome variable while measuring muscle quality in response to the intervention in both groups. The small sample size may have contributed to the current findings. Persons with complete motor SCI suffer from limited mobility; however, the findings may have been influenced by the level of injury (i.e., paraplegia or tetraplegia) as well as the level of physical activity on a daily basis. The pilot nature of the work should be considered with caution, especially with the failure to attain statistical significance. The heterogeneity of injury levels and completeness among participants are also important confounding variables. Therefore, self-reported spasticity measurement may provide an important clinical implication, as participants may have different levels of spasticity prior to admission and following intervention. The current work has limited power and future clinical trials are highly needed to confirm or refute the observed trends following either intervention.

## 6. Conclusions

Despite the limited power, electrically evoked muscle hypertrophy did not exacerbate knee extensor or flexor spasticity, or alter the dosages of anti-spastic medications in persons with SCI. The findings may support a potential neuroprotective or modulatory effect of TT on either the extensor spasticity slopes or catch slopes. However, the exact mechanism has yet to be explored. Additionally, the findings may highlight the permanent effects of training on spasticity. The use of an isokinetic dynamometer provides a safe and objective assessment of different components of spasticity in individuals with SCI. Future clinical trials with larger sample sizes including both males and females are warranted to examine the effects of TT with and without exercise on spasticity in persons with SCI.

## Figures and Tables

**Table 1:** Physical and SCI characteristics of participants who were randomized into either 16 weeks of NMES-RT+TT or TT only.

Group	ID	Gender	Age (yrs)	Ethnicity	Weight (kg)	Height (cm)	BMI (kg/m^2^)	TSI (yrs.)	LOI	Class.	ASIA Class.
NMES-RT+TT	001	M	49	AA	107.5	188.6	30.0	24	T4	Para	A
	004	M	45	AA	90.5	183.2	27.0	14	T4	Para	A
	005	M	48	W	70.5	166.5	25.4	28	T4	Para	A
	014	M	34	W	74.8	178.3	23.5	14	T8-T9	Para	B
	017	M	19	W	69.0	174.8	22.6	2	T10	Para	A
	021	M	49	AA	81.5	168.2	28.8	6	T6	Para	B
	025	M	33	W	105.5	182.7	31.5	3	C5	Tetra	A
	Mean ± SD		40 ± 11	3 AA: 4 W	86 ± 16	177.5 ± 8.0	27.0 ± 3.4	13 ± 10	C5-T10	6 Para: 1Tetra	5A:2B
TT only	002	M	49	AA	79.2	171.36	27.0	4	C6	Tetra	B
	003	M	40	AA	60.5	185.55	17.6	14	C6	Tetra	B
	008	M	30	AA	88.0	180.5	27.0	4	T6	Para	A
	012	M	34	W	62.3	179.8	19.3	6	C6	Tetra	A
	016	M	31	W	76.7	175.9	24.8	7	T5	Para	A
	022	M	27	W	80.5	181.56	24.4	2	C6	Tetra	A
	Mean ± SD		35 ± 8	3 AA: 3 W	74.5 ± 11	179 ± 5.0	23.5 ± 4.0	6 ± 4	C6-T6	2 Para: 4 Tetra	4A:2B

M: male; AA: African American; W: White.

**Table 2:** Modified Ashworth scores of the knee extensor muscle group, antispastic medications and the dosages for participants in both groups.

Group	ID	Modified Ashworth Scores of Knee Extensors	Anti-Spastic Medications	Dosages (mg)
NMES-RT+TT	001	1	Baclofen	20
	004	1	None	-
	005	0	None	-
	014	3	None	-
	017	4	None	-
	021	0	Baclofen	10
	025	2	Baclofen	10
	Mean ± SD	1.6 ± 1.6		13 ± 6
TT only	002	3	Baclofen	10
	003	2	None	-
	008	1	None	-
	012	3	None	20
	016	0	None	20
	022	1	Baclofen	20
	Mean ± SD	1.8 ± 1.3	Baclofen	17.5 ± 10
Grades	Description			
0	No increase in muscle tone			
1	Slight increase in muscle tone with slight catch and release or minimal resistance at end of stretch			
1+	Slight increase in muscle tone with minimal resistance after catch that lasts throughout remainder of range of motion			
2	Moderate increase in muscle tone but affected part still easily moved			
3	Considerable increase in muscle tone with difficulty in passive range of motion			
4	Affected part is rigid in flexion (bent) or extension (straight)			

## Data Availability

Data will be available upon contacting the corresponding author, A.S.G., and upon obtaining necessary approval from the ethical and research committee of the Richmond VA Hospital.

## Supplementary Materials

The following supporting information can be downloaded at: https://www.mdpi.com/article/10.3390/jcm14113972/s1, Table S1: Mean extensor and flexor spasticity with 95% CI at different angular velocities after randomized into either NMES-RT+TT or TT only groups.

## Abbreviations

ANOVA	analysis of variance
ANCOVA	analysis of covariance
BL	baseline measurements
BMI	body mass index
CSA	cross-sectional area
DXA	dual energy x-ray absorptiometry
MAS	Modified Ashworth scale
MRI	magnetic resonance imaging
NMES-RT	neuromuscular resistance training
PI	post-intervention measurements
RT	resistance training
SCI	spinal cord injury
TT	testosterone treatment
