# Supplementary material for: Role of Electrically Evoked Muscle Hypertrophy on Spasticity in Persons with Spinal Cord Injury
Source: J Clin Med. 2025 Jun 4;14(11):3972. doi: 10.3390/jcm14113972 (PMC12155623; doi:10.3390/jcm14113972)
Supplement: Supplementary file 1 [file jcm-14-03972-s001.zip › jcm-3631328-supplementary.pdf]

Suppl Table 1. Mean extensor and flexor spasticity with 95% CI at different angular velocities after randomized into either NMES-RT+TT or TT only groups.

	Angular Velocities (deg/sec)	5		30		60		90		180		270	
		**BL**	**PI**	**BL**	**PI**	**BL**	**PI**	**BL**	**PI**	**BL**	**PI**	**BL**	**PI**
**Extensor Spasticity**	NMES-RT+TT-	6.6±2.0	7.3±1.2	8.0±1.2	8.2±1.7	9.2±1.7	8.8±1.7	8.4±1.25	8.9±1.9	9.0±1.6	9.5±1.8	7.9±2.2	9.2±1.5
	**95% CI**	5.25-8.0	6.2-8.4	7.2-8.9	7.0-9.4	7.8-10.5	7.5-10.2	7.2-9.6	7.6-10.4	7.5-10.5	7.9-11.0	6.1-9.7	7.4-11.1
	TT only-	6.6±0.8	6.4±1.3	6.9±0.8	6.9±1.2	8.4±1.3	8.2±1.5	8.1±1.7	8.4±1.4	8.7±2.0	8.6±1.8	8.5±1.9	8.4±3.0
	**95% CI**	5.0-8.2	5.1-7.7	6.0-7.8	5.6-8.3	7.0-9.8	6.7-9.6	6.8-9.4	6.9-9.9	7.1-10.3	7.0-10.3	6.4-10.6	6.2-10.6
**Flexor Spasticity**	NMES-RT+TT-	7.2±1.6	7.2±3	6.15±1.7	6.7±0.98	5.0±2.1	5.9±1.5	4.5±2.6	4.9±0.92	4.2±2.1	4.9±2.0	4.2±2.1	4.4±1.3
	**95% CI**	6.0-8.3	5.2-9.1	5.0-7.3	5.7-7.6	3.5-6.5	4.5-7.3	2.7-6.4	3.4-6.4	2.6-5.9	3.2-6.5	2.6-5.9	3.2-5.6
	TT only-	6.1±1.2	6.3±1.0	5.6±0.86	5.9±1.3	4.5±1.2	5.4±1.8	4.6±1.4	5.2±2.1	4.5±1.8	4.6±2.0	4.0±1.8	3.6±1.6
	**95% CI**	4.8-7.4	4.2-8.4	4.4-6.8	4.8-6.9	2.9-6.1	3.9-6.9	2.7-6.4	3.7-6.6	2.7-6.2	2.8-6.4	2.0-5.9	2.1-5.0

CI: confidence interval; **BL:** baseline; **PI:** post-intervention
